# Learning-based robotic grasping: A review

**DOI:** 10.3389/frobt.2023.1038658

**Published:** 2023-04-04

**Authors:** Zhen Xie, Xinquan Liang, Canale Roberto

**Affiliations:** ^1^ Advanced Remanufacturing and Technology Centre (ARTC), Agency for Science, Technology and Research (A*STAR), Singapore, Singapore; ^2^ Singapore Institute of Manufacturing Technology (SIMTech), Agency for Science, Technology and Research (A*STAR), Singapore, Singapore

**Keywords:** versatile grasping, learning policy, high mix and low volume, personalization, tactile sensing, soft gripping

## Abstract

As personalization technology increasingly orchestrates individualized shopping or marketing experiences in industries such as logistics, fast-moving consumer goods, and food delivery, these sectors require flexible solutions that can automate object grasping for unknown or unseen objects without much modification or downtime. Most solutions in the market are based on traditional object recognition and are, therefore, not suitable for grasping unknown objects with varying shapes and textures. Adequate learning policies enable robotic grasping to accommodate high-mix and low-volume manufacturing scenarios. In this paper, we review the recent development of learning-based robotic grasping techniques from a corpus of over 150 papers. In addition to addressing the current achievements from researchers all over the world, we also point out the gaps and challenges faced in AI-enabled grasping, which hinder robotization in the aforementioned industries. In addition to 3D object segmentation and learning-based grasping benchmarks, we have also performed a comprehensive market survey regarding tactile sensors and robot skin. Furthermore, we reviewed the latest literature on how sensor feedback can be trained by a learning model to provide valid inputs for grasping stability. Finally, learning-based soft gripping is evaluated as soft grippers can accommodate objects of various sizes and shapes and can even handle fragile objects. In general, robotic grasping can achieve higher flexibility and adaptability, when equipped with learning algorithms.

## 1 Introduction

Robotic grasping is an area of research that not only emphasizes improving gripper design that can handle a wide variety of objects but also drives advances in intelligent object recognition and pose estimation algorithms. Grasping objects differ in terms such as weight, size, texture, transparency, and fragility factors. To achieve efficient robotic grasping, the collaboration and integration of mechanical and software modules play a pivotal role, which also opens several possibilities for enhancing the current state of the art of robotic grasping technology. For instance, tactile feedback from gripper fingertips can serve as a valid input for grasping decision makers (Xie et al., 2021) to determine the grasping stability, and visual servoing can correct the grasping misalignment (Thomas et al., 2014).

**FIGURE 1:**
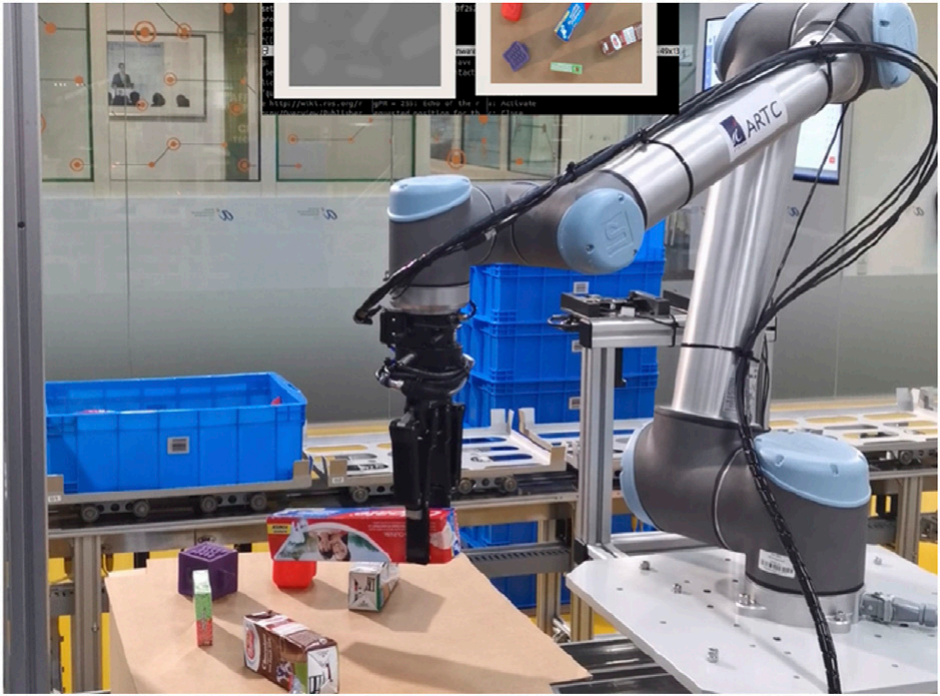
Robotic grasping in the cluttered FMCG scenario.

This paper provides insights into industries such as manufacturing, logistics, or fast-moving consumer goods (FMCG) that face challenges after the adoption of pre-programmed robots. These robots require reprogramming when new applications are needed, thus being suitable only for limited application scenarios. This results in pre-programmed robots to be inadequate for fast-changing processes. Moreover, most of the solutions are unable to pick or grasp novel unknown objects in a high-mix and low-volume (HMLV) production line, as shown in Figure 1. These high-mixed SKUs include various types of products, for example, heavy, light, flat, large, small, rigid, soft, fragile, deformable, and translucent.

**FIGURE 2:**
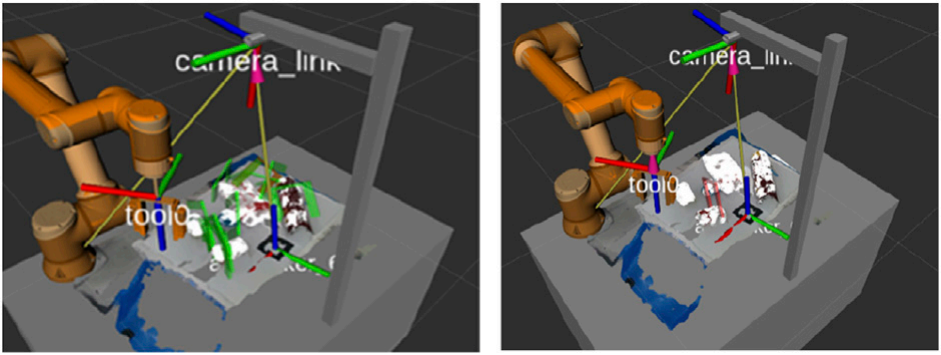
**(A)** Grasping pose candidates by sampling. **(B)** Final grasping pose with the maximum score.

In general, grasping can be applied to three types of objects: familiar, known, and unknown (Bohg et al., 2013). Known objects mean the objects that have been included in the training previously and the grasping pose has been generated and executed by the robots for the grasping motion. On the contrary, unknown objects and familiar objects are never encountered previously, but familiar objects have a certain similarity with training datasets. Grasping for known objects has been implemented in the industry for quite some time as the technology is mature when compared to the other two categories. The challenge lies in grasping familiar or unknown objects with minimum training or reconfiguration required. The research and development focus on transferring the grasping motion from known objects to familiar or unknown objects based on the interpretation and synthetic data (Stansfield, 1991; Saxena et al., 2008; Fischinger and Vincze, 2012). Based on the grasping evaluation metric, grasping with the best scores would be selected among all the grasping candidates, as shown in Figure 2.

Grasping poses can be ranked by similarities in the grasping database. Moreover, because of its high difficulty, current research is chiefly focused on developing deep learning (DL) models for grasping unknown objects, with some prominent ones utilizing deep convolutional neural networks (DCNNs), 2.5D RGBD images, and depth images of a scene (Richtsfeld et al., 2012; Choi et al., 2018; Morrison et al., 2018). These methods are generally successful in determining the optimal grasp of various objects, but they are often restricted by logistical issues such as limited data and testing.

**FIGURE 3:**
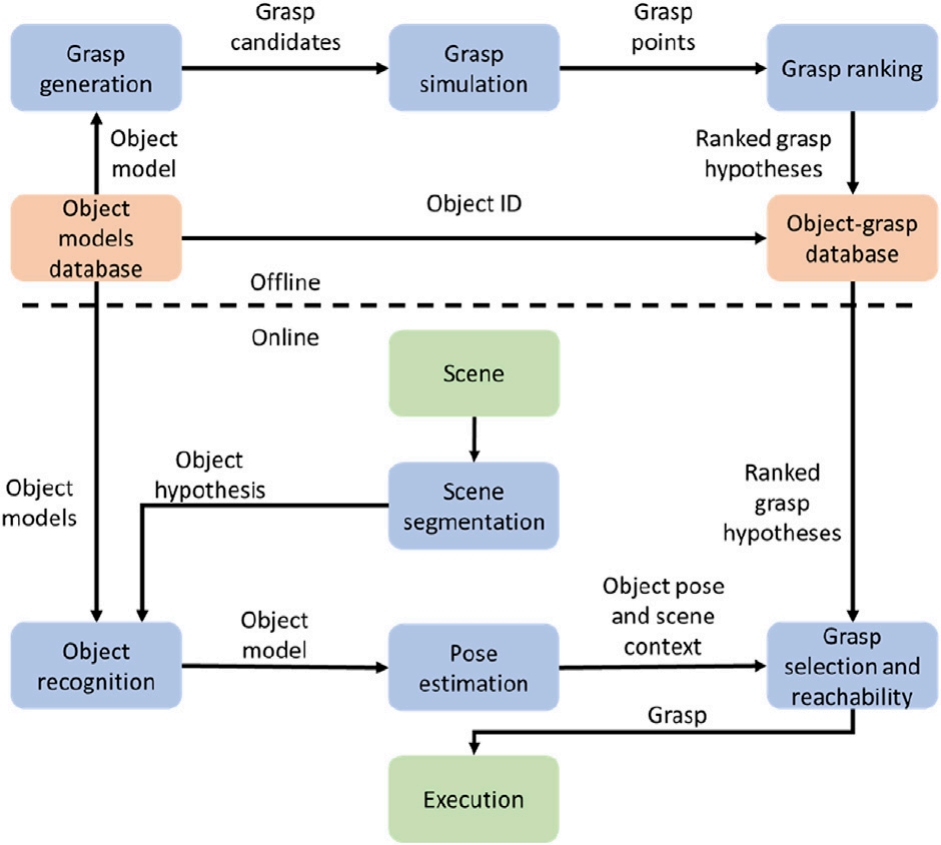
Online and offline processes of grasping generation (Bohg et al., 2013).

The flow chart of a general grasping process, including offline generation and online grasping, is demonstrated in Figure 3. In the offline phase, the training was conducted on grasping different objects. Moreover, the quality is evaluated for each grasping process. After that, the grasping model is generated based on the training process and stored in the database. Moreover, in the online phase, the object is detected through vision and mapped to the model database. A grasping pose is generated from the learning database, and those objects that cannot be grasped are discarded. Finally, the grasping motion is conducted by the robot.

In this paper, we reviewed over 150 papers on the topic of intelligent grasping. We categorize the literature into six main categories. To be more specific, 3D object recognition, grasping configuration, and grasping pose detection are some typical grasping sequences. Deep learning and deep reinforced learning are also reviewed as widely used methods for grasping and sensing. As one of the trending gripping technologies, soft and adaptive grippers with smart sensing and grasping algorithms are reviewed. Finally, tactile sensing technologies, which enable smart grasping, are reviewed as well. The search criteria used were the following:

• *Year:* 2010 has been selected as the cutoff year such that the bulk of the papers reflect the last dozen years. A few exceptions before 2010 were included due to their exceptional relevance.
• *Keywords: “*3D object recognition”, “robotic grasping”, “learning based”, “grasping configuration”, “deep learning”, “grasping pose detection”, “deep learning for unknown grasping”, “soft gripper”, “soft grippers for grasping”, “tactile sensors”, and “tactile sensors for grasping” were the keywords used.
• *Categories:* paper selection requirement included belonging to one of the following overarching categories:
  o Robotic grasping
  o Robotic tactile sensors
  o Soft gripping
  o *Learning-based approaches*

Results were filtered out based on individual keywords or key word combinations, using the AND and OR operand between keywords. Moreover, Google Scholar, IEEE Xplore, and arXiv were deployed as the main sources of search engine for the literature in both journals and conference proceedings between 2017 and 2022. In total, excluding duplicates, we found 329 papers close to the unseen object grasping theme, out of which 157 had the full text available. In Section 2, we conduct a review on the most significant contributions and developments in robotic grasping, soft grippers, and tactile sensors for grasping. Section 3 contains an analysis of the challenges for learning-based approaches for grasping; Section 4 summarizes our findings.

**FIGURE 4:**
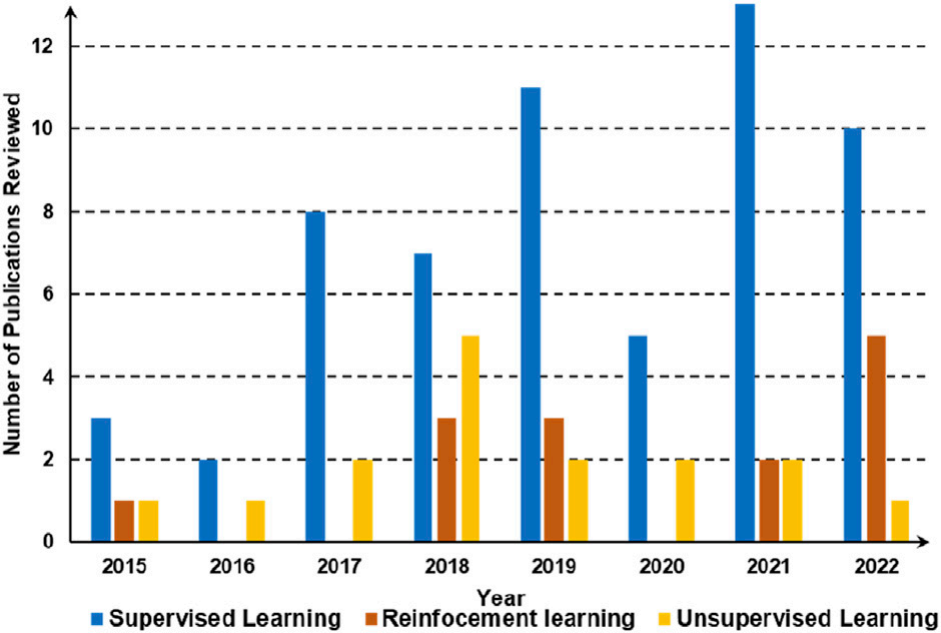
Number of publications of learning algorithms for intelligent grasping in recent years.

Figure 4 demonstrates the trend in the number of publications of learning algorithms for intelligent grasping in recent years. The graph shows the growth in the number of published works from this literature review in the field of intelligent grasping with a focus on the three major approaches, namely, supervised learning, reinforcement learning, and unsupervised learning. The data covers the period from 2015 to 2022, demonstrating the increasing interest and advancements in the development of intelligent grasping algorithms; in particular, since 2017, there has been a significant increase in supervised learning, unsupervised learning, and reinforcement learning. However, supervised learning is still the most adopted approach for AI-driven robotic grasping.

## 2 Methods and recent developments

A. 3D object recognition benchmarks

The first step for the grasping sequence is to identify the grasping object. 2D learning-based object recognition has been well developed in computer vision. Thus, in this section, we will focus on the advances on 3D learning-based object recognition. Traditionally, the object point cloud can be segmented from the environment based on region growing (Vo et al., 2015) and the Point Cloud Library (PCL) (Zhen et al., 2019). After that, principal component analysis (PCA) (Abdi and Williams, 2010) can be deployed to identify the centroid point of the object along the eigenvector (Katz et al., 2014), which can be used as inputs for robotic grasping. Next, the Iterative Closest Point (ICP) (Besl and McKay, 1992; Chitta et al., 2012) is also a popular approach to registering the available model into the point cloud to locate the object for grasping. However, the disadvantage is the need for tuning excessive hyperparameters. Concerning benchmarks such as ImageNet, ResNet-50, and AlexNet (Dhillon and Verma, 2020), two parallel DCNNs can be deployed to extract multimodal features from RGB and depth images, respectively (Kumra and Kanan, 2017). The same theory is applied to other enhanced 3D approaches, for example, 3D Faster R-CNN (Li et al., 2019), 3D Mask R-CNN (Gkioxari et al., 2019), and SSD (Kehl et al., 2017).

With the advances of big data, there are many 3D object benchmarks emerging where either point clouds were collected or labeled, such as PointNet (Qi et al., 2017a), PointNet++ with deep hierarchical feature learning (Qi et al., 2017b), BigBird (Zaheer et al., 2020), Semantic3D (Hackel et al., 2017), PointCNN (Li et al., 2018a), SpiderCNN (Xu et al., 2018), Indoor inference NYUD-V2, and Washington RGB-D Object Dataset (Lai et al., 2011), or 3D model datasets were gathered with information such as textures, shapes, hierarchies, weight, and rigidity, for example, ShapeNet (ChangFunkhouser et al., 2015), PartNet (Mo et al., 2019), ModelNet (Wu et al., 2015), and YCB (Calli et al., 2015). Other approaches apply convolution to the voxelization of point clouds VoxNet (Maturana and Scherer, 2015) and Voxception-ResNet (Brock et al., 2016). However, high memory and computational costs are key drawbacks associated with 3D convolutions. Specifically, a segmentation algorithm can be built upon these datasets to separate and locate the object in the clustered environment.

Overall, point cloud-based approaches perform more efficiently when the raw point cloud input is sparse and noisy (Shi et al., 2019). Moreover, it can reduce data preprocessing time since raw point clouds can be used directly as inputs and object identification is omitted, so efforts for sampling, 3D mesh conversion, and 3D registration are saved. Most notably, CAD data might not be available all the time. However, point cloud datasets can lose information that is critical for grasping, such as textures, materials, and surface normals. Topology needs to be recovered in order to improve the representation of the point cloud (Wang et al., 2019).

B. Grasping configuration sampling benchmarks

Learning for object recognition is not enough for robotic manipulation. The subsequent step relies on the grasping pose estimation (Du et al., 2021) based on the gripper configuration. In particular, grasping perception can be treated as analogous to traditional CV object detection (Fischinger et al., 2013; Herzog et al., 2014) with RGBD or point clouds as inputs. First, a grasping region of interest (ROI) is sampled and identified; next, a large number of grasping poses can be generated based on big training datasets without knowing object identification (Kappler et al., 2015). This approach works well for novel objects; however, the success rate is not reliable enough to be implemented in the real-world scenarios. Template matching using the convex hull or bounding box is another grasping pose detection method (Herzog et al., 2012).

Except for the large-scale data collection and empirical grasp planning in physical trials directly (Levine et al., 2018), there are plenty of grasping benchmarks that contain sizable numbers of grasping datasets, which can be categorized into different groups based on the grasping technology or gripper configuration. GraspNet (Fang et al., 2020), SuctionNet (Cao et al., 2021), DexYCB (Chao et al., 2021), OCRTOC (Liu et al., 2021), the Columbia Grasp Database (Goldfeder et al., 2009), Cornell dataset (Jiang et al., 2011), off-policy learning (Quillen et al., 2018), TransCG (Fang et al., 2022) for transparent objects, and Dex-net (MahlerLiang et al., 2017; Mahler et al., 2018) are the most prominent benchmarks. Furthermore, Dex-net 4.0 trained ambidextrous policies for a parallel jaw and a vacuum-based suction cup gripper. Though learning-based grasping detection still needs handcrafted inputs to generalize to unknown objects (Murali et al., 2018), methods such as multiple convolutional neural networks (CNNs) (Lenz et al., 2015) in a sliding window detection pipeline are proposed to address the issues.

**FIGURE 5:**
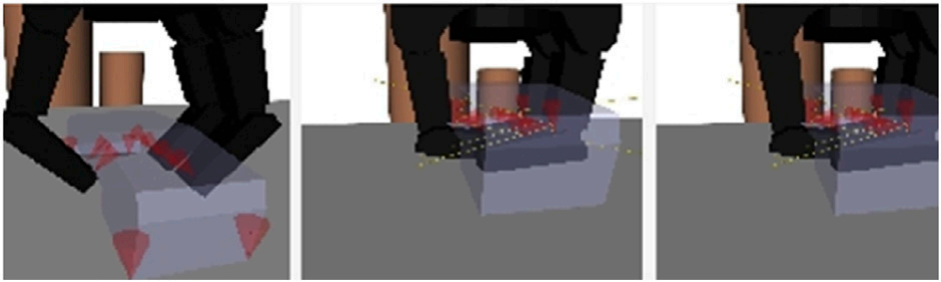
GraspIt simulator for pose prediction (Miller and Allen, 2004).

In contrast, GraspIt (Miller and Allen, 2004) utilizes a simulator to predict the grasping pose; however, fidelity in a simulation environment is still far from the real world, as demonstrated in Figure 5. Domain randomization (Tobin et al., 2017) can be the key to transfer the learning of simulated data. GraspNet (Fang et al., 2020) is a popular open project for general object grasping that is continuously enriched. There are 97,280 3D images in total and each image is annotated with an accurate 1.1 billion 6D poses for each object, and 190 cluttered scenes were captured using Kinect A4Z and RealSense D435. Moreover, Jiang et al. (2011) proposed a method for the oriented grasping rectangle representation that considers the seven-dimensional gripper configuration and uses it for fast search inference in the learning algorithm. The limitation is that the grasping diversity is affected by the rectangular configuration, and the grasping area is restrained.

Among the most popular grasping pose sampling benchmarks, the literature reports a 91.6% prediction accuracy rate and an 87.6% grasping success rate on selected grasping objects for the Cornell datasets (Jiang et al., 2011), while GraspNet has been shown to attain a success rate of 88% on a range of objects with diverse appearances, scales, and weights that are frequently used in daily life (Mousavian et al., 2019). Dex-Net 4.0 claims a reliability of over 95% for 25 novel objects. Previously, the GQ-CNN-based Dex-Net 3.0 showed a precision of 99% and 97% for the basic and typical objects in the dataset (Mahler et al., 2018), respectively. PointNet 40-class classification has 89.2% accuracy rate using the ModelNet40 compared to 85.9% by VoxNet and 84.7% by 3D ShapeNets (Qi et al., 2017a). Last but not least, using the same datasets, SpiderCNN achieves an accuracy of 92.4% on standard benchmarks while PointNet++ reaches 91.9% (Xu et al., 2018).

C. Grasping pose evaluation (GPE)

Grasping pose evaluation is the selection process to find the most suitable grasping candidate based on the specific evaluation metrics after grasping pose sampling. Many non-learning-based grasping pose evaluation metrics have been developed, such as SVM ranking model analysis-by-synthesis optimization (AbS) (Krull et al., 2015), kernel density estimation (Detry et al., 2011), and robust grasp planning (RGP) (MahlerLiang et al., 2017), as have other physics-based approaches such as force closure (Nguyen, 1988), caging (Rodriguez et al., 2012), and Grasp Wrench Space (GWS) analysis (Roa and Suárez, 2015). Only recently, learning-based approaches have been proposed, such as variational autoencoders (VAE) for DL (Mousavian et al., 2019; Pelossof et al., 2004), the cross-entropy method (CEM) for RL (De Boer et al., 2005), random forest (Asif et al., 2017a), grasp quality convolutional neural network (GQ-CNN) (Mahler et al., 2018), deep geometry-aware grasping network (DGGN) (Yan et al., 2018), grasp success predictor based on deep CNN (DCNN), and dynamic graph CNN (Wang et al., 2019). Moreover, we have also seen trends of the fusion of classic approaches with deep learning (empirical), such as AbS combined with deep learning for reliable performance on uncontrolled images (Egger et al., 2020), cascaded architecture of random forests (Asif et al., 2017a), and a supervised bag-of-visual-words (BOVW) model with SVM (Pelossof et al., 2004) or AdaBoost (Bekiroglu et al., 2011).

**FIGURE 6:**
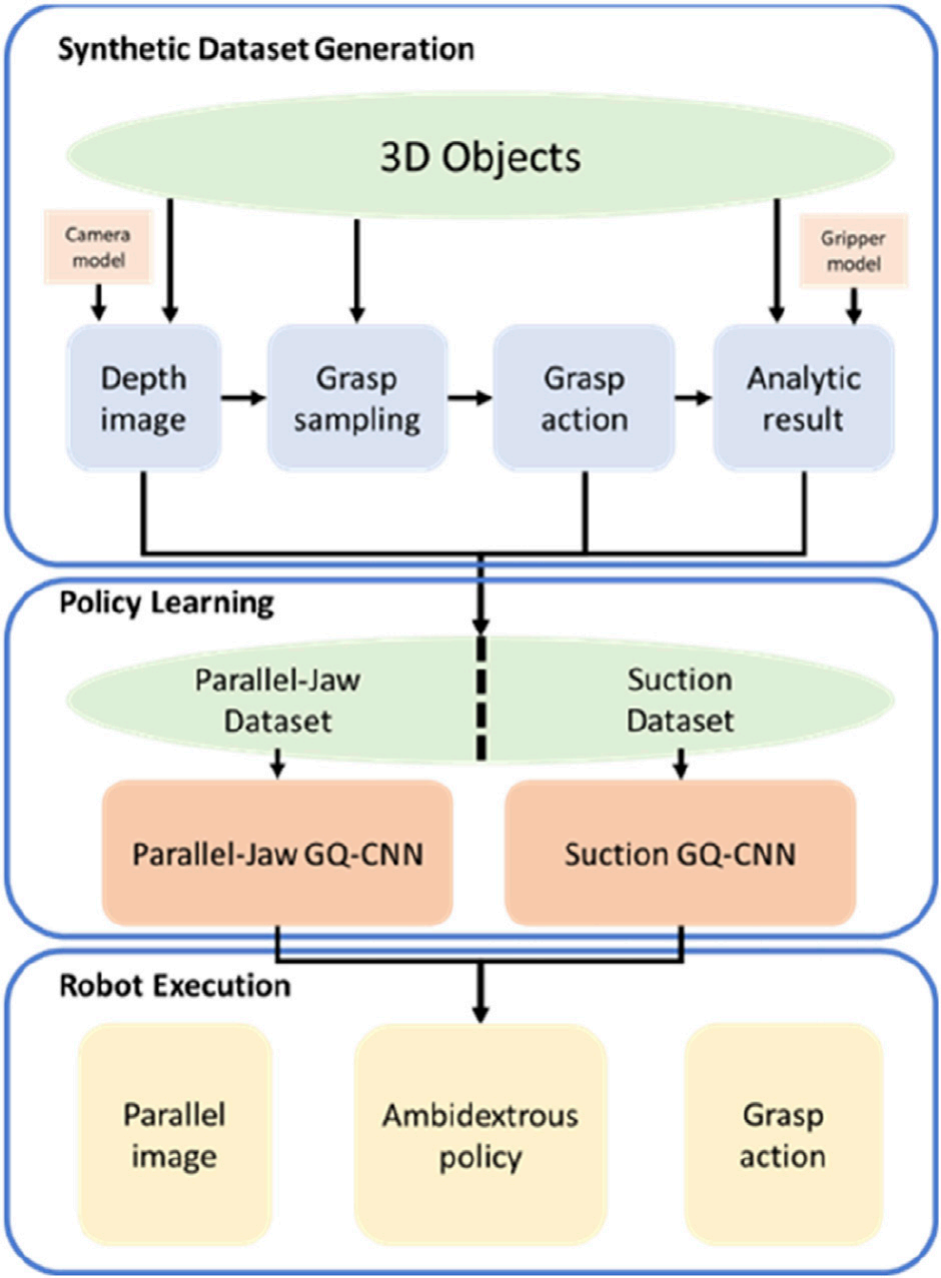
Ambidextrous grasping policy learning (Mahler et al., 2019).

The schematic of the ambidextrous grasping policy-learning process is shown in Figure 6. Synthetic 3D-object datasets are generated *via* computer-aided design (CAD) with some domain randomization. The generated objects are tested in the synthetic training environment to evaluate the rewards, which are computed consistently based on the resistance to grasping. In terms of policy learning, parallel jaw and suction grippers are trained by optimizing a deep GQ-CNN to predict the probability of grasp success from the point cloud of the 3D CAD model objects. The training dataset contains millions of synthetic examples from the previous generation step. Furthermore, for robot execution, the ambidextrous policy is adopted by a real-world robot to select a gripper to maximize the grasp success rate using a separate GQ-CNN for each gripper.

Grasping Pose Detection (GPD) (ten Pas et al., 2017) utilizes a four-layer CNN-based grasp quality evaluation model. Even though the heuristic produces diverse grasping candidates, the limitation is that the GPD might mistake multiple objects as one due to a lack of object segmentation, and the GPD might have overfitting problems when the point cloud is sparse.

Similarly, PointNetGPD (Liang et al., 2019) introduces lightweight network architecture by the point cloud within a gripping finger that is transformed into a local grasp representation. The orthogonal approaching and parallel moving directions are along the ZXY axes, respectively, with the origin lying at the bottom center of the gripper. The grasping quality is evaluated by *N* points that are passed through the network.

**FIGURE 7:**
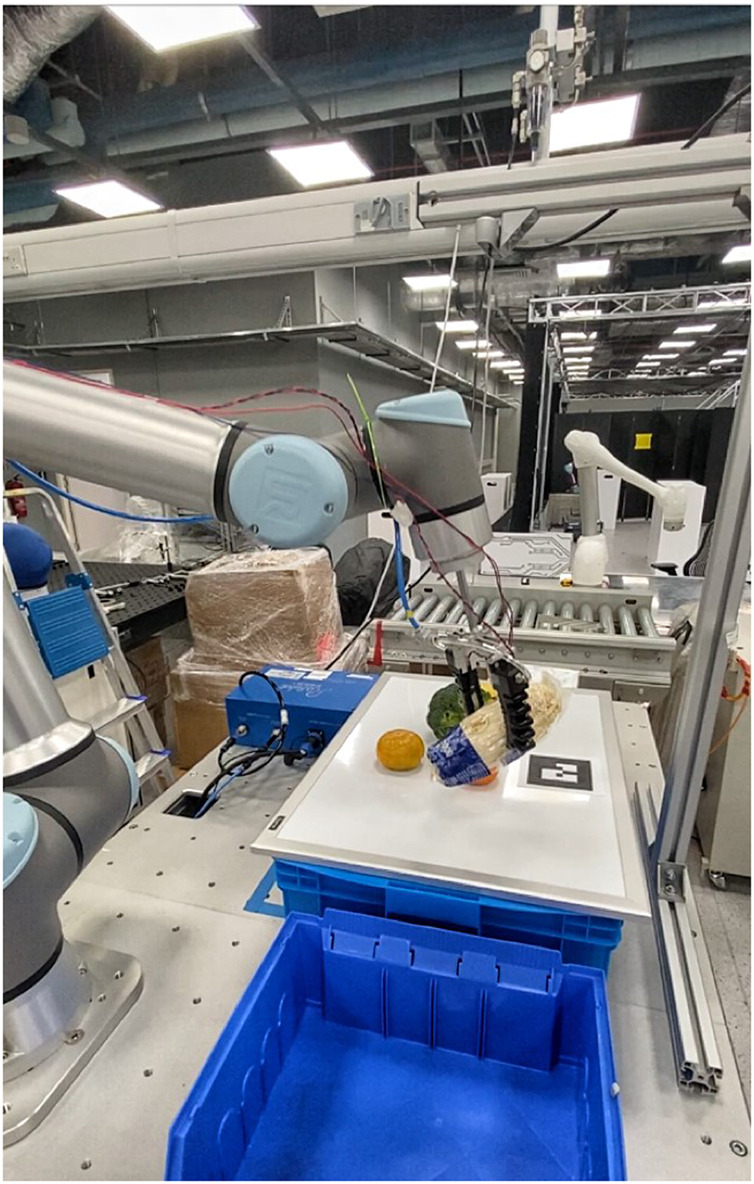
Data-driven soft gripping on the novel object.

Xie et al. (2022) proposed a universal soft gripping method with a decision maker based on tactile sensor feedback on objects with varying shapes and textures, which is a further improvement from the PointNetGPD baseline (Liang et al., 2019). Figure 7 shows the grasping of enoki mushroom that is unknown to the training databases.

Table 1 shows the comparison table between various grasping pose evaluation methods in terms of inputs, grasping type, specifications, and learning type in the recent literature. If force (force closure) or wrench (GWS) is taken into consideration, only grasping hand or finger grippers can be used for this type of application. However, force closure requires tactile sensor reading to be more accurate and real time in order to be practical (Saito et al., 2022). Moreover, parametric GPE such as SVM can be applied if the grasping shape can be represented or estimated by parameters. SVM, random forest, and supervised bag-of-visual-words are utilized for supervised or self-supervised learning applications only. When a large number of data are presented, data-driven learning-based methods such as GPD and DGGN are more suitable to make sense of big data and perform better than other types of GPE methods. Last but not least, ensemble learning methods, such as AdaBoost, have gained more attention recently, which combine multiple learning methods to provide better evaluation results (Yan et al., 2022).

D. Reinforcement learning (RL) approach

**TABLE 1:** Learning-based grasping pose evaluation.

Grasping pose evaluation	Metric type	Input	Grasping type	Technical specification	Learning/type
Yu et al. (2021a)	Support vector machine (SVM)	Superquadric shape parameters and grasping parameters	Hands, grippers, and suction	Difficult to represent complex shapes in superquadric parameters and vector sets as alternatives	Regression and supervised learning
Yen-Chen et al. (2021) and Irshad et al. (2022)	Analysis-by-synthesis optimization (AbS)	3D textured reconstruction	Hands, grippers, and suction	Complex multi-object scenarios and learned latent space	Regression
Katyara et al. (2021)	Kernel density estimation	Object-relative grasp poses	Hands, grippers, and suction	Continuous probability density functions, non-parametric	Non-parametric regression
MahlerLiang et al. (2017) and Zhang et al. (2021)	Robust grasp planning (RGP)	Prior grasps and 3D object models	Hands, grippers, and suction	Correlated bandit techniques and cloud-based object models	Multi-armed bandit and CNN
Liu et al. (2022a)	Force closure	Object and gripper pose, contact, and friction	Hands, grippers, and soft	Reduces the complexity and universal, force spiral space, and binary	N/A
de Souza et al. (2021)	Grasp wrench space analysis (GWS)	Contact location, contact normal, and frictional coefficients (Weisz and Allen, 2012)	Hands, grippers, and soft	Epsilon quality and magnitude of the minimum norm wrench	Deep learning
Wang et al. (2022a)	Variational autoencoder (VAE)	Primitive grasp set with the generated grasp set and gripper configuration	Hands, grippers, and suction	Compressed representation, Kullback–Leibler (KL) divergence, and latent space sampling	CNN and machine learning
HuangNagaraj et al. (2021)	Random forest	Grasp features	Hands, grippers, and suction	Quantified as its Gini impurity-based importance can be used for deformable grasping	Supervised machine learning
Jiang et al. (2022)	Grasp quality convolutional neural network (GQ-CNN)	Point clouds, grasps, and analytic grasp metrics MahlerLiang et al. (2017)	Hands, grippers, and suction	Grasp features represented as the angle, planar position, and depth of a gripper relative to an RGB-D camera	CNN
Wang et al. (2022b)	Deep geometry-aware grasping network (DGGN) Yan et al. (2018)	Point cloud, shape, location, and orientation	Parallel jaw grippers	Shape generation network and grasping outcome prediction network	Deep Learning and 3D CNN
Mi et al. (2021) and Liang and Boularias (2022)	Dynamic graph CNN	Segmented depth and color image	Hands, grippers, and suction	Generalizes to new objects with different geometries and textures	CNN
Yu et al. (2021b)	Cascaded architecture of random forests Asif et al. (2017a) and Asif et al. (2017b)	RGB-D point clouds	Hands, grippers, and suction	Object-class and grasp-pose probabilities are computed, separated, and fused for unknown objects	CNN
Ayoobi et al. (2022)	Supervised bag-of-visual-words	Scene data	Hands, grippers, and suction	Uses local feature descriptors to match database Ergene and Durdu (2017)	Supervised learning
Miften et al. (2021)	AdaBoost	Object shape, grasp information, tactile information, and gripper configuration	Hands, grippers, and suction	Probabilistic learning framework, capable of inferring based on tactile measurement	Ensemble learning
Jiang et al. (2021)	Grasping pose detection (GDP)	Point cloud and gripper configuration	Hands, grippers, and suction	Directly on the point cloud w/o estimating grasping pose, can be used in the clustered environment	Deep learning

RL does result in flexible and more adaptable robotic grasping algorithms. Policy gradient methods, model-based methods, and value-based methods are the three most popular deep reinforcement learning methods (Arulkumaran et al., 2017). However, value-based learning such as Q-learning has the limitation of optimization on a non-convex value function, thus making it difficult for large-scale RL tasks until scalable RL with stochastic optimization over the critic was proposed to avoid second maximizer networks (Kalashnikov et al., 2018). These algorithms can be further divided into two categories: off-policy learning and tactile feedback. Off-policy learning (Quillen et al., 2018) is emphasized and generalized to unseen objects. Common off-policy learning methods include Point Cloud Library (PCL) (Nachum et al., 2017), deep deterministic policy gradient (DDPG) methods (LillicrapHunt et al., 2015), deep Q-learning (MnihKavukcuoglu et al., 2013), Monte Carlo (MC) policy evaluation (Xie and Zhong, 2016a; Arulkumaran et al., 2017), and more robust-corrected Monte Carlo methods (Betancourt, 2017).

Human-labeled data are intrinsically subjective due to human bias, which is a problem of increasing concern in machine learning (Xie et al., 2017; Fonseca et al., 2021). Researchers have attempted to adopt unsupervised learning by trial and error for object manipulation learning from scratch (Boularias et al., 2015) but were restrained by a small amount of data. Pinto and Gupta (2016) trained CNN using large-scale datasets and proved that multi-stage training can get rid of overfitting problems for self-supervised robotic grasping (Zhu et al., 2020).

Self-supervised learning (Berscheid et al., 2019) that connects manipulation primitive shifting with prehensile action grasping based on Markov decision processes (MDPs) can significantly improve grasping in clustered scenarios. Some other prominent works, such as Visual Pushing and Grasping (VPG) (Zeng et al., 2018), utilize two fully convolutional networks trained by self-supervised Q-learning for inference of pushes and grasps, respectively, based on the sampling of end effector orientation and position. The limitation is that only simple push and grasping motions are considered among all the non-prehensile manipulation primitives, and the grasping objects demonstrated are regular shapes. Finally, motion primitives are pre-defined, and alternative parameterizations are needed to improve the motion expressiveness. Multi-functional grasping (Deng et al., 2019) with a deep Q-Network (DQN) can improve the successful grasping rate from the clustered environment by tagging the performance of suction gripping.

Our review involved comparing data-driven approaches with deep reinforcement learning (DRL) approaches (Kalashnikov et al., 2018), and it revealed that the limitations of DRL such as being data-intensive, complex, and collision-prone, preventing itself from being industry ready.

E. Chronological map

**FIGURE 8:**
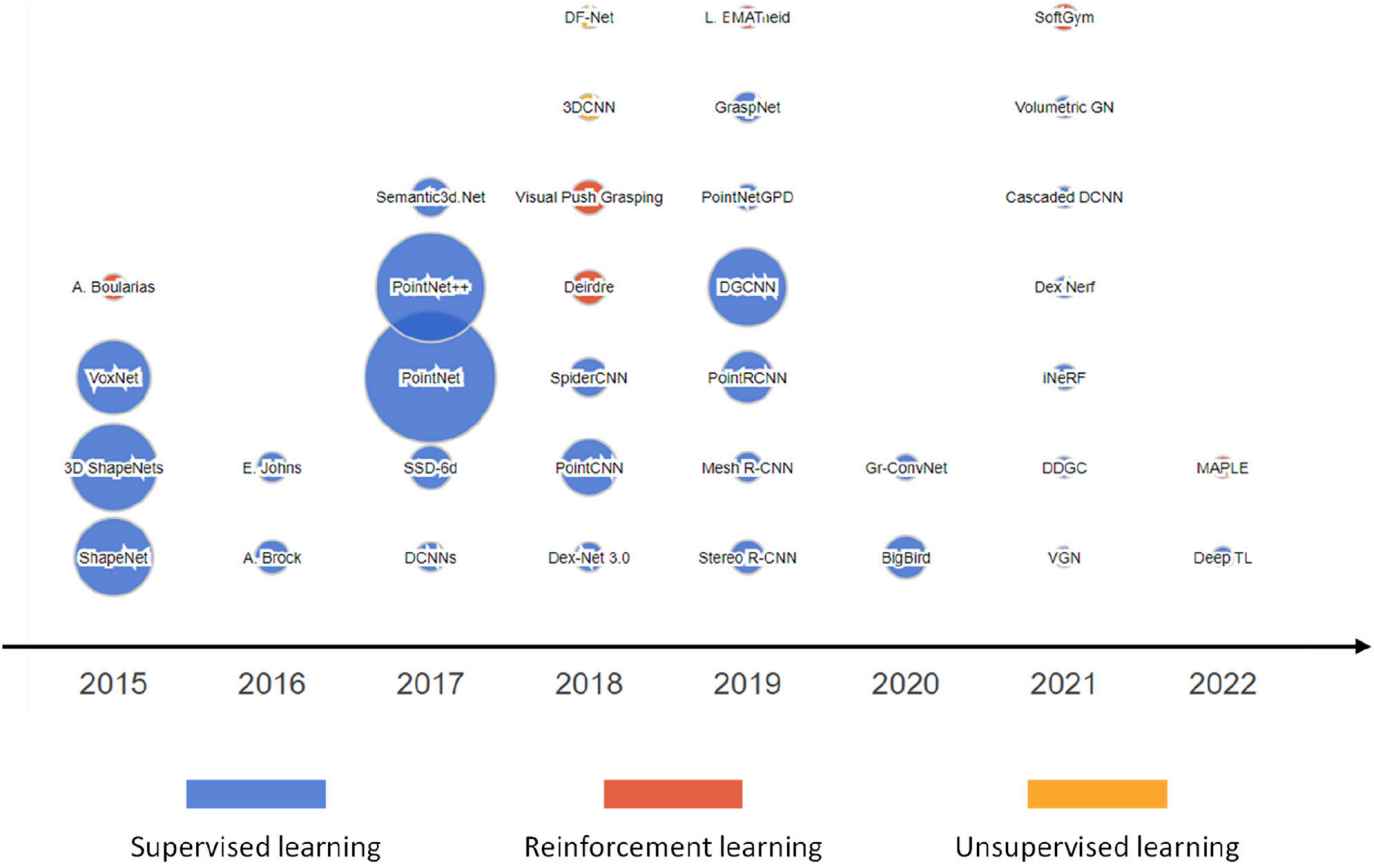
Key learning algorithms for intelligent grasping.

Figure 8 presents the development of key learning algorithms for intelligent grasping from 2015 to 2022. The data show the popularity of three main categories of algorithms, namely, supervised learning, reinforced learning, and unsupervised learning. The size of each bubble represents the number of research papers published in each category, while the *x*-axis indicates the year of publication. Some popular algorithms include PointNet, PointNet++, and 3D ShapeNets. The figure indicates that supervised learning is still the major algorithm used for intelligent grasping. Reinforced learning and unsupervised learning are also obtaining more attention in recent years.

F. Soft gripping technology

As an alternative to traditional rigid grippers, soft grippers have been researched and developed in the last decade (Rus and Tolley, 2015; Hughes et al., 2016; Shintake et al., 2018; Whitesides, 2018). Due to the intrinsic softness and compliance of gripper materials and actuation mechanisms, the control complexity is greatly reduced for handling delicate objects and irregular-shaped objects. The early studies on soft grippers mainly focused on soft materials, structure design optimization, and actuating mechanisms. In recent years, control strategies and smart grasping are becoming more essential in soft gripper research. Learning algorithms are adopted in soft grippers to enable intelligent grasping. The objective of using learning algorithms mainly falls into two domains: object detection/classification during grasping and increasing grasping success rates.

To enable object detection/classification when grasping using soft grippers, the sensors are usually integrated on the soft fingers to perceive the grasping mode. To detect the deformation of each finger, strain sensors are implemented into the soft grippers (Elgeneidy et al., 2018; Jiao et al., 2020; Souri et al., 2020; Zuo et al., 2021). These sensors can detect the deformation of bending actuators so that the grasping pose of different fingers can be estimated. Long short-term memory (LSTM) (Xie and Zhong, 2016b) is typically utilized to process the data and classify the objects from the SoftMax function (Zuo et al., 2021). However, since the strain sensors are normally made for one axis detection, they are usually insufficient for detection and need to be used together with other sensors such as tactile (Zuo et al., 2021) and vision (Jiao et al., 2020) sensors. Tactile sensors are widely used for detecting objects (Jiao et al., 2020; She et al., 2020; YangHan et al., 2020; Subad et al., 2021; Zuo et al., 2021; Deng et al., 2022). They can be built and fabricated on a small scale and embedded into soft grippers. Tactile sensors can be made from capacitance sensing (Jiao et al., 2020; Zuo et al., 2021), optical fiber-based sensing (YangHan et al., 2020), microfluidics, (Deng et al., 2022), or even vision-based tactile sensors (She et al., 2020). Since the grasping motion dynamically changes with time, LSTM is usually adopted to process the data and classify the objects. Other kinds of sensors, such as IMU (Della Santina et al., 2019; Bednarek et al., 2021), can be used to detect the motion of the soft gripper to estimate the grasping process. LSTM is used for IMU sensors (Bednarek et al., 2021), but CNN-based methods, such as YOLO v2, are applied if vision is used together with IMU (Della Santina et al., 2019).

Vision-based learning algorithms are widely used to train the grasping mode and increase the grasping success rate. De Barrie et al. (2021) proposed a study on using CNN to capture the deformation of an adaptive gripper so that the stress on the gripper can be estimated to detect the grasping motion. Yang et al. (2020) used a fully convolution neural network (FCNN) to detect whether the grasping was successful, and the grasping data were based on the soft–rigid, rigid–soft, and soft–soft interaction. Liu et al.( 2022b) used double deep Q-learning (DDQN)-based deep reinforced learning to train a multimodal soft gripper for employing different grasping modes (grasping or vacuum suction) for different objects. Wan et al. (2020) used CNN to detect the objects and benchmark the effectiveness of using different finger structures (three or four fingers; circular or parallel) for object grasping. Zimmer et al. (2019) integrated accelerometer, magnetometer, gyroscope, and pressure data on the soft gripper and used RealSense to detect the objects. Different learning algorithms, including support vector machine (SVM), Spatio-Temporal Hierarchical Matching Pursuit (ST-HMP), FFNN, and LSTM, were compared in this study based on their sensor structures.

To conclude this, the combination of soft grippers with learning algorithms is still a new research field, and the papers have mainly been published in the past 4 years. The compliant properties of the soft gripper eases the concerns regarding grasping delicate objects, while object detection/classification and grasping mode optimization is the key research field. LSTM-based learning algorithms are widely used for object detection/classification during grasping, and CNN-based algorithms are used for vision-based learning for increasing grasping success rates.

G. Tactile sensors for robotic grasping

Tactile feedback is an alternative area for off-policy learning. Wu et al. (2019) achieved grasps by coarse initial positioning of the multi-fingered robot hand.

The maximum entropy (MaxEnt) RL policy is optimized through Proximal Policy Optimization (PPO) with a clipped surrogate objective to learn exploitation and exploration (E/E) strategies. The robot can decide the grasping recovery and whether to proceed with a re-grasp motion based on the proprioceptive information.

Tactile sensing technology has been rapidly developing in the past few years with strong interest from the research community. Tactile sensors are classified according to their physical properties and how they acquire data: capacitive, resistive, piezoelectric, triboelectric, ultrasonic, optical, inductive, and magnetic (Wang et al., 2019), (Baldini et al., 2022), (Dahiya and Valle, 2008). Traditionally used in the medical and biomedical industry for prosthetic rehabilitation or robotic surgery applications (Al-Handarish et al., 2020), tactile technology is now common in robotics. Grasping and manipulation tasks exploit tactile sensors for contact point estimation, surface normals, slip detections, and edge or curvature measurements (Kuppuswamy et al., 2020), (Dahiya and Valle, 2008), while recent applications for physical HRI are proposed by Grella et al. (2021). These sensors can provide dense and detailed contact information, especially in occluded spaces where vision is unreliable. However, these sensing capabilities can be worsened by external object compliance (Kuppuswamy et al., 2020).

Traditional low-cost off-the-shelf force-sensitive resistor tactile sensors (Tekscan, 2014) are still used as tactile sensors to provide end-of-arm tools (EOAT) with force sensing capabilities. Current research studies, however, show different trends and design principles when developing new tactile technologies. These can be summarized as follows:

• *Minimal and resilient design* (Subad et al., 2021)*,* (Kuppuswamy et al., 2020): low power, simple wiring, minimal dimensions, single layers, durable, and resistant to stress (mechanical and shear).
• *Distributed* (Jiao et al., 2020)*,* (Cannata et al., 2008)*,* (Kuppuswamy et al., 2020): expandable, flexible, conformable, and spatially calibrated.
• *Information dense* (Elgeneidy et al., 2018; Xie and Zhong, 2016b; Yuan et al., 2017; Jeremy et al., 2013): high resolution, multimodal sensing, and multi-dimensional contact information.

These design principles are extracted from the current state-of-the-art tactile sensing technology. Novel and established tactile sensors are summarized in Table 2, which highlights each sensor’s technological features and their use in machine learning for grasping.

H. Deep learning *via* tactile technology

**TABLE 2:** Learning-based tactile sensing.

Sensor	Sensor type	Sensing capability	Size/hardware	Technical specification	Learning/type	Advantage	Limitation
Digit (Lambeta et al. 2020), 2020	Optical	Tactile images	22 × 27 × 18 mm, camera, elastomer gel, and RGB LEDs	60 fps, 1.15 mm FoV, 300 mm DoF, and USB	ResNet-18, 3D Conv Lambeta et al. (2021)	Cheap, fast, adaptable to multiple applications	Bulky rigid case, no force sensing, and limited surface
HEX-O-SKIN (Mittendorfer and Cheng (2011), 2011	Thermal, MEMS accelerometer, and Optical	Pressure, proximity, vibration, orientation, temperature, Thermal flow	3.6 mm thickness and 5.1 cm^2^ area hard hexagonal patches	2g, 1kHz, distributed, and UART	N/A	Multiple sensing capabilities, customizable, and distributed	Bulky and proprietary
CySkin (Cannata et al. (2008), 2008	Capacitive	Digital capacitance	25 cm^2^ area, flexible PCB, and capacitive taxels	5–10Hz, distributed CAN BUS, SPI, and USB	HandsNet, CNN (Lambeta et al. (2021)	Customizable surface cover, high resolution, distributed, and flexible	Expensive, requires spatial and pressure calibration, and proprietary
Punyo (Kuppuswamy et al. (2020), 2020	Optical, depth	3D contact point cloud and contact shape	86 × 88 × 172.5 mm, camera, ToF sensors, and soft compliant dotted latex membrane	10 k points, 1 Hz	N/A	High resolution, 2D and 3D information, open source, and compliant surface	Bulky and requires special adaptors
GelSight (Yuan et al. (2017), 2017	Optical	Tactile images, 3D Surface Shape, and force	Variable dimensions, camera, elastomer gel surface, and RGB LEDs	1–100 µm spatial resolution, and USB	DNN (LSTM + CNN) (Li et al. (2018b)	Force sensing and high resolution	Bulky and limited surface
BioTac (Jeremy et al. (2013); Reinecke et al. (2014), 2006	Impedance sensing, and thermal	Impedance, AC/DC pressure, micro vibrations, temperature, thermal f low, and force	Rigid core, conductive fluid, elastometric skin, electrodes, thermistor	3.2 mV, 36.5 Pa, 0.37 Pa, 0.1 °C, 0.001°C/s resolution, withstand up to 50N, UDP, PCAN-PCI, and SPI USB	Tactile GCN, CNN, and LSTM (Garcia-Garcia et al. (2019); Mi et al. (2021)	Multiple sensing capabilities	Complex installation procedure and limited surface
FlexiForce (Tekscan (2014)	Piezoresistive	Force	.02 mm thickness and variable area	3% accuracy, −40 °C–60 °C, up to 30kN, and USB	N/A	Flexible, distributed, and force sensing	Commercial
(Kim et al. (2020), 2020	Air pressure	Pressure array	Silicone base and air pressure sensing module	1Pa resolution, distributed, I^2^C, and CAN BUS	CNN	Distributed	Complex installation procedure
(Tenzer et al. (2014)), 2014	Barometric	Pressure array	5 × 3 × 1.2 mm and MEMS transducer covered by rubber	50–115 kPa range, 0.01 N sensitivity, distributed, I^2^C, and USB	SVM (Wan et al. (2016)	Distributed	High precision and force saturation
FingerVision (Zhang et al. (2018), 2018	Optical	Tactile image	Fish-eye camera and elastomer gel with markers	15 FPS	ConvLSTM, LSTM (Zhang et al. (2018); Zhang et al. (2020)	High resolution and deformable	Bulky and limited surface

Deep learning and neural networks have successfully attempted to use tactile data as input and feedback in grasping and manipulation stability evaluation. Sensors such as GelSight (Yuan et al., 2017) or DIGIT (Lambeta et al., 2020) are already supported by open-source software packages to simulate, test, and train grasping and manipulation. TACTO (Wang et al., 2022c) is a PyBullet-based open-source simulator that is able to reproduce and render tactile contacts, learn manipulation tasks, and reproduce them in the real world. PyTouch (Lambeta et al., 2021) is a machine learning library to process tactile contacts and provides built-in solutions such as contact detection. Tactile sensors and modern machine learning techniques are used to solve grasp stability, control, contact detection, and grasp correction. Contact models, grasp stability, and slip detections are learnable outputs that can be generalized to novel objects for dexterous grasping (Kopicki et al., 2014). Schill et al. (2012) and Cockbum et al. (2017) used tactile sensors mounted on a robotic hand for bin picking which were able to generate a tactile image that is fed to SVM algorithms, achieving between 70% and 80% grasping stability in both papers. Wan et al. (2016) had a similar set-up and used another SVM prediction from tactile contact to classify grasp outcomes. Li et al. (2018b) mounted pressure sensors on a three-finger gripper and used an SVM for stability prediction; despite the 90% accuracy, the limited performance of SVMs is acknowledged in the previous papers, and the authors encourage the use of more complex algorithms. Grella et al. (2021) used a tactile skin for an industrial pHRI application gripper by human detection *via* a simple DNN called HandsNet. Wan et al. (2016) proposed various LSTM-based DNNs and Pixel Motion to predict contact detection from tactile images generated from the FingerVision sensor, achieving 98.5% accuracy. Kim et al. (2020) used a simple DNN to linearize tactile information, which was then used to optimize the proposed torque control scheme. Calandra et al. (2018) compared tactile only and vision + tactile information to improve grasping; tactile and visual images were fed to ResNet, and multi-layer perceptron evaluated grasp success stability. Li et al. (2018b) proposed a similar architecture with the GelSight tactile sensor + vision on pre-trained networks with LSTM and an FC layer to detect slip during grasping, showing how multimodal inputs can, in general, improve grasp stability and avoid slippage. A graph convolutional network acquiring tactile data from the BioTac was proposed in both Garcia-Garcia et al. (2019) and Mi et al. (2021) to predict grasp stability; this method can, in general, outperform LSTM and SVM, but both papers show that the higher the graph connectivity, the lower the accuracy.

## 3 Trends and challenges

In this section, we discuss the trends and challenges on grasping benchmarks, tactile sensors, and learning-based soft gripping.

A. Learning-based grasping pose generation

Several 3D grasping sampling benchmarks emerged with the help of learning-based 3D segmentation benchmarks. The current development of learning-based grasping pose generation provides advantages of adaptability to novel objects and various gripper configurations. However, the success rate is still not reliable enough to be implemented in the real world, and manual feature engineering is still needed to generalize to unknown objects. We can see trends of the fusion of traditional approaches with an empirical approach to address the grasping quality evaluation. Grasping from the clustered environment remains a challenge as non-prehensile primitive actions (Zeng et al., 2018) are involved to decouple the occluded objects. Moreover, multimodal perception data (Saito et al., 2021) have been used besides vision to provide broader coverage regarding the grasping stability. However, grasping tagged on reinforcement learning demonstrates the tendency to become computationally lightweight, free from overfitting, simplified, and more collision aware. Last but not least, how to enrich training datasets using synthetic simulation data still remains a research challenge.

B. Tactile sensors and grasping

The literature shows that tactile sensors are being developed with *Minimal*, *Resilient*, *Distributed* and *Information Dense Design* as guiding principles (Cannata et al., 2008; Mittendorfer and Cheng, 2011; Zimmer et al., 2019; Wan et al., 2020). This is to improve the hardware and software implementation and provide meaningful information regarding the contact. The main limitations of these sensors are bulky designs, complex integration, and costs. The main advantage is in providing rich multimodal information in an occluded situation or when the visual input is not sufficient. Vision-based tactile sensors are becoming increasingly popular as tactile images are a rich kind of information that can be successfully used in machine learning.

Grasping is intrinsically variable due to variations in the target pose and position, the grasping hardware and software, and the external environment (Wan et al., 2016). Adopting successful grasping policies is the challenge that tactile machine learning is successfully attempting to solve. SVM is a typical approach that has been successfully implemented to predict grasping stability (Schill et al., 2012; Wan et al., 2016; Cockbum et al., 2017; Li et al., 2018b) with acceptable accuracy but limited generalizability. Traditional CNNs have been proposed in Li et al. (2018b), Calandra et al. (2018), and Lambeta et al. (2021) for various applications, such as tactile image classification, which can be useful in various scenarios. Novel and efficient use of GCN is shown in Zhang et al. (2018) and Zhang et al. (2020) despite limited generalizability and a trade-off in size and accuracy. LSTM networks have been successfully proposed for grasp detection, stability, and slip detection (Schill et al., 2012; Li et al., 2018b; Zhang et al., 2018; Zhang et al., 2020) in various scenarios and applications. CNNs have also been used together with LSTM for grasping stability (Garcia-Garcia et al., 2019). Overall, the review shows that LSTM is the most promising class of DNNs for tactile sensors, as sequences of tactile images provide more insightful and usable data. There is a little known work on contact wrenches and torsional and tangential force interpretation (Zhang et al., 2020) with either DNNs or traditional algorithms. This has a high potential to improve grasping stability and force-closure estimation.

Overall, machine learning and tactile technologies are still being heavily researched; however, tactile sensors are becoming cheaper and more readily available, and a few valid design principles and trends have been identified such as *Minimal and Resilient Design*, *Distributed*, and *Information Dense*. On the other hand, ML applications with tactile technology are still at an exploratory stage, with no clear dominant market trend or approach. This shows that the technology is still not yet industry ready and is quite immature, which leaves room for further research and improvement toward more reliable and accurate solutions.

C. Learning-based soft gripping

Deep learning and deep reinforced learning have dominated the recent research to train the soft grippers for successful grasp and object detection. However, the soft grippers used for training are usually not state-of-the-art design architectures. Cable-driven underactuated soft grippers and adaptive soft grippers are still the trends in this field. In the future, a more functional soft gripper with versatile grasping capabilities should be used for smart grasping operations. Furthermore, most of the research employed very mature algorithms, such as LSTM, for tactile-based sensing and CNN for vision-based sensing. The development of more specific algorithms for soft grippers is necessary to fully utilize the advantages of the soft grippers. Moreover, to extend the sensing capabilities and enable more precise grasping, various sensors, such as force sensors, strain sensors, and vision systems, need to be further developed and integrated into the soft grippers. With more features from the sensors, object detection can have higher accuracy.

## 4 Conclusion

In this study, we first conducted a literature survey on data-driven 3-day benchmarks and grasping pose sampling algorithms. After that, grasping evaluation metrics and deep learning-based grasping pose detection were discussed. The comparison results showed that the learning-based approach performs quite well in terms of grasping unknown objects. In terms of the success rate of grasping, the current learning-based methods fail to achieve a reliable percentage for real-world-ready products and are not yet ready for production line deployment. Finally, we did see trends in the development of tactile sensors and soft gripping technology to improve grasping stability. Some recent work has been carried out on learning-based grasping with tactile feedback, and we could see that more compatible robotic sensors have emerged. A clear finding is that a successful and effective solution is the combination of the right problem statement with suitable hardware and the proper AI-enabled algorithm. With the findings regarding the current technologies and research trends, the current challenges of learning-based grasping pose generation, tactile sensing, and soft gripping are proposed. We expect future works will focus on multimodal deep learning with various supplementary grasping proprioceptive and exteroceptive information.
